# Deep Learning for Semantic Segmentation vs. Classification in Computational Pathology: Application to Mitosis Analysis in Breast Cancer Grading

**DOI:** 10.3389/fbioe.2019.00145

**Published:** 2019-06-21

**Authors:** Gabriel Jiménez, Daniel Racoceanu

**Affiliations:** ^1^Sciences & Engineering Faculty, Pontificia Universidad Católica del Perú, Lima, Peru; ^2^Engineering Department, Pontificia Universidad Católica del Perú, Lima, Peru; ^3^Faculty of Science & Engineering, Sorbonne University, Paris, France

**Keywords:** deep learning, CNN, semantic segmentation, mitosis detection, mitosis segmentation, whole slide imaging, digital pathology, computational pathology

## Abstract

Existing computational approaches have not yet resulted in effective and efficient computer-aided tools that are used in pathologists' daily practice. Focusing on a computer-based qualification for breast cancer diagnosis, the present study proposes two deep learning architectures to efficiently and effectively detect and classify mitosis in a histopathological tissue sample. The first method consists of two parts, entailing a preprocessing of the digital histological image and a free-handcrafted-feature Convolutional Neural Network (CNN) used for binary classification. Results show that the methodology proposed can achieve 95% accuracy in testing, with an F1-score of 94.35%. This result is higher than the results using classical image processing techniques and also higher than the approaches combining CCNs with handcrafted features. The second approach is an end-to-end methodology using semantic segmentation. Results showed that this algorithm can achieve an accuracy higher than 95% in testing and an average Dice index of 0.6, higher than the existing results using CNNs (0.9 F1-score). Additionally, due to the semantic properties of the deep learning approach, an end-to-end deep learning framework is viable to perform both tasks: detection and classification of mitosis. The results show the potential of deep learning in the analysis of Whole Slide Images (WSI) and its integration to computer-aided systems. The extension of this work to whole slide images is also addressed in the last sections; as well as, some computational key points that are useful when constructing a computer-aided-system inspired by the proposed technology.

## 1. Introduction

Recent estimations by the World Health Organization positioned breast cancer in the top 5 killing types of cancer in the world (DeSantis et al., 2015; World Health Organization, 2018). Only in the U.S, nearly 330 080 new cases of breast cancer (invasive and non-invasive) and 40 920 deaths were reported in 2018 (Breastcancer.org U.S., 2018). Due to the remarkable technological advances in breast cancer imaging, mammography is currently the non-invasive reference screening technique for early detection of breast cancer. Nevertheless, histological examination of tissue specimens remains the gold standard for diagnosis and accurate evaluation of breast diseases (Owens and Ashcroft, 1987).

In order to objectively classify the tumor and determine the treatment strategy for patients with breast cancer, pathologists often use the Nottingham Histology Score (NHS), also referred to as the Scarff-Bloom-Richardson grading system (Bloom and Richardson, 1957). NHS assigns a score from 1 to 3 to the tissue sample in three categories: (i) presence of glandular/tubular structures; (ii) nuclear pleomorphism; and (iii) mitotic count. The sum of all these scores will determine the cancer grading and its related severity. Due to their different stages and forms, mitotic counting is one of the most challenging and time-consuming tasks for pathologists. The present article focuses on this particular challenge, one of the key features for an accurate diagnosis.

### 1.1. Digital Pathology

Digital systems were introduced to the histopathological examination in order to deal with the huge and complex amount of information obtained from tissue specimens. Digital images were originally generated by mounting a camera on the microscope. The static images captured only reflected a small region of the glass slide, and the reconstruction of the whole glass slide was not frequently attempted due to its complexity and time-consuming characteristics. However, precision in the development of mechanical systems has made possible the construction of devices such as whole slide digital scanners. The high-resolution images so generated, allow pathologists to view, manage, and analyze the digitized tissue on a computer monitor, in a similar manner as under an optical microscope.

Whole slide imaging (WSI) technology, also initially referred to as *virtual microscopy*, have proven to be useful in a wide variety of applications in pathology (e.g., image archiving, telepathology, image analysis). In essence, a WSI scanner operation principle consists in moving the glass slide a small distance every time a picture is taken, in order to capture the entire tissue sample. Every WSI scanner has six components: (a) a microscope with lens objectives, (b) light source (bright field and/or fluorescent), (c) robotics to load and move glass slides around, (d) one or more digital cameras for capture, (e) a computer, and (f) software to manipulate, manage, and view digital slides (Pantanowitz et al., 2015). The hardware and software used for these six components will determine the key features to analyze when choosing a scanner. The Digital Imaging and Communications in Medicine (DICOM) standard was adopted to store WSI digital slides into commercially available PACS (Picture Archiving and Communication System) and facilitate the transition to digital pathology in clinics and laboratories. Due to the WSI dimension and size, a pyramidal approach for data organization and access was proposed by the DICOM Standards Committee, Working Group 26 (2010). In Pantanowitz et al. (2015), Farahani et al. compared 11 WSI scanners from different manufacturers regarding imaging modality, slide capacity, scan speed, image magnification, image resolution, digital slide format, multi-layer support, and special features their hardware and software may offer. This study showed that robotics and hardware used in a WSI scanner are currently state-of-the-art and almost standard in every device. Software, on the other hand, has some ground for further development.

### 1.2. Computational Pathology

Computational pathology is a term which refers to the integration of WSI technology and image analysis tools, in order to perform tasks that were too cumbersome or even impossible to undertake manually. Image processing algorithms have evolved yielding enough precision to be considered in clinical applications. A few examples mentioned in Pantanowitz et al. (2015) include morphological analysis to quantitatively measure histologic structures (Kong et al., 2013); automated selection of regions of interest such as areas of most active proliferative rate (Lu et al., 2014); and automated grading of tumors (Ho et al., 2014). Moreover, educational activities have also benefited from the development of computational pathology. Virtual tutoring, online medical examinations, performance improvement programs, and even interactive *illustrations* in articles and books are being implemented thanks to this technology (Pantanowitz et al., 2015).

In order to validate a WSI scanner for clinical use, several tests are conducted following the guidelines developed by the College of American Pathologists (CAP). On average, reported discrepancies between digital slides and glass slides are in the range of 1 to 5%. However, even glass-to-glass slide comparative studies can yield discrepancies due to observer variability and increasing case difficulty (Pantanowitz et al., 2015).

Although several studies in the medical community have reported using WSI scanners to perform the analysis of tissue samples, pathologists remain reluctant to adopt this technology in their daily practice. Lack of training, limiting technology, shortcomings to scan all materials, cost of equipment, and regulatory barriers have been pointed as the principal issues (Pantanowitz et al., 2015). In fact, it was until early in 2017 that the first WSI scanner protocol was approved by the FDA (ESMO, 2017). Therefore, WSI technology has now the full potential to enhance the practice of pathology by introducing new tools which help pathologists provide a more accurate diagnosis, based on quantitative information.

## 2. Literature Review

Microscopy has evolved remarkably over the years by incorporating imaging processing techniques. In the last decade, it has also benefited from the integration of artificial intelligence (AI) algorithms which have been shown to improve diagnostic accuracy and provide quantitative metrics useful for pathologists. In fact, in 2018, researchers at Google AI Healthcare reported the integration of modern AI into a standard microscope to detect metastatic breast cancer in sentinel lymph nodes and prostate cancer in prostatectomy specimens (Chen et al., 2018). Efforts made in this field are frequently driven by the need to overcome financial and workflow barriers encounter when using whole slide imaging scanners (e.g., prices of WSI scanners, IT infrastructure, operating personnel, among other). However, due to the advantages the latter technology poses, several researchers have been studying different alternatives to integrate AI and image processing algorithms with WSI.

### 2.1. Detection and Classification of Cell Nuclei in Histological Images

Operator-bias in cancer grading is undoubtedly one of the most important problems of cancer diagnosis and grading. In particular, nuclei analysis is the most cumbersome task for pathologists due to its different properties and representations in a digital image. Regarding breast cancer and mitosis analysis, the image processing algorithms studied in the literature are categorized into two different groups: segmentation and classification.

Detection and segmentation of mitosis have been extensively studied in the literature. In Paul and Mukherjee (2015), the authors suggested two different subcategories for segmentation algorithms: region based cell segmentation and boundary based cell segmentation. Among region-based approaches, Yang et al. proposed a novel marker-controlled watershed algorithm which can effectively segment clustered cells with fewer over-segmentation Yang et al. (2006). Nedzved et al. also applied morphological operations, and combined them with thinning algorithms to segment cells in histological images Nedzved et al. (2000). In order to improve robustness, different nuclear models using different morphological features were proposed and validated by Lin et al. (2007). Moreover, in Paul and Mukherjee (2015), authors proposed a segmentation controlled by the relative entropy between cells and background using opening and closing morphological operations. Similarly, Chowdhury et al. applied entropy thresholding to detect and segment monocyte cells in order to track them using bipartite graph matching algorithms (Chowdhury et al., 2010). Contextual information from objects in an image was also reported as a methodology for detection and segmentation of cell nuclei. Seyedhosseini et al. introduced a framework called multi-class multi-scale series contextual model, which uses contextual information from multiple objects and at different scales for learning discriminative models in a supervised setting Seyedhosseini and Tasdizen (2013). Following the model of extracting information from several scales, Al-Kofahi et al. proposed an automatic segmentation algorithm using graph-based binarization and multi-scale Laplacian-of-Gaussian filtering (Al-Kofahi et al., 2010). On the other hand, regarding the second subcategory, level set methods are quite popular in the literature for boundary-based segmentation (Mukherjee et al., 2004; Nath et al., 2006; Dzyubachyk et al., 2008). Active contour was also reported in Lee et al. (1999) for automated segmentation of breast cancer nuclei. To sum up, most region-based methodologies assume a similarity in the size or region properties of different cells which is not the case for mitosis in breast cancer. Meanwhile, boundary-based algorithms are strongly dependent on the initial conditions and stopping criteria. Either way, all algorithms rely on prior knowledge for detection and segmentation which does not allow the development of fully automated systems.

Literature for cell nuclei classification is as extended as the one reported for segmentation. Sertel et al. proposed pixel level likelihood functions and component-wise two-step thresholding for mitosis counting in digitized images. They reach an average sensitivity of 81.1 and 12.2% of false positive detections (Sertel et al., 2009). Unsupervised clustering algorithms were also studied by Roullier et al. In Roullier et al. (2011), authors proposed a graph-based multi-resolution approach which yielded a good sensitivity (≥70%) for Grade 1 and Grade 3 breast cancer in WSI. Similarly, Weyn et al. proposed a k-nearest neighbor classification approach combined with wavelets features for multiscale image analysis of isolated nuclei in invasive breast cancer tissue. Authors achieved a recognition score of 76%; however, the presence of false negative cases restricted its immediate practical use (Weyn et al., 1998). Other machine learning approaches were also considered in the literature. Irshad et al. evaluated different classifiers (decision tree, linear kernel SVM and non-linear kernel SVM) using color and texture features (e.g., blue ratio, Haralick, HMAX, and SIFT), and reached 76% in f-score (Irshad et al., 2013). Tao et al. also studied SVMs using 59 parameters combining geometric properties and intensity information. Results showed an 89.2% in accuracy using a specific subset of features (Tao et al., 2007). An ensemble of cascade adaboosts (Tek, 2013) was also reported to yield a significant improvement in mitosis classification. However, this method fails to demonstrate its robustness as region features used in the study may have significant variations among various datasets. Genetic algorithms for mitosis classification were proposed by Nateghi et al. (2014). The authors used genetic optimization algorithms to eliminate potential non-mitosis from the histological image. Then, texture features were computed from the remaining potential cell nuclei and classify using SVMs. Results were promising (78.47% f-score); however, they did not make a considerable improvement in the machine-learning category for mitosis classification. Taking a step further, the authors in Dalle et al. (2008) proposed the first complete grading system of breast cancer using histological images. In particular, for mitosis detection, they proposed two Gaussian models for classification using geometric and intensity features. The results obtained were promising; however, the system's scores tend to be slightly lower than pathologist's scores. With the development of artificial intelligence algorithms for image analysis, a few studies were conducted regarding the mitosis classification task. In Cireşan et al. (2013), authors proposed a deep max-pooling convolutional neural network approach for mitosis classification which achieved 0.72 f-score. Recently, Saha et al. improve the performance of convolutional neural networks (CNN) by adding additional information from hand-crafted features (Saha et al., 2018). Results showed a promising 0.9 f-score which demonstrated the out-performance of deep-neural-network approaches over classical machine-learning ones. Araujo et al. also contributed to the study of CNN by proposing a methodology which uses the features obtained from the convolutional layers as inputs of a support vector machine (SVM) classifier. This method achieved an 83.3% accuracy and 95.6% sensitivity (Araújo et al., 2017). Although these last results suggest there are better ways to address the mitosis challenges in histological imaging, the lack of available ground-truth data is still a major issue to fully incorporate deep learning into the biomedical imaging domain. Albarqouni et al. evaluated a method combining crowd-sourcing for data annotation and convolutional neural networks for classification. However, this proposal needs further research as the results reported do not exceed 0.8 in f-score (Albarqouni et al., 2016). To sum up, Table 1 summarizes and categorizes the methodologies exposed in the last paragraphs according to the image analysis algorithms employed.

**Table 1:** Summary of the literature review.

**Tasks**	**Categories**	**Methods proposed**
Mitosis detection/segmentation	Region-based algorithms	Marker control watershed (Yang et al., 2006), Morphology (Nedzved et al., 2000; Lin et al., 2007), Entropy thresholding (Chowdhury et al., 2010), Contextual model (Seyedhosseini and Tasdizen, 2013), Graph binarization (Al-Kofahi et al., 2010).
	Boundary-based algorithms	Level sets (Mukherjee et al., 2004; Nath et al., 2006; Dzyubachyk et al., 2008), Active contours (Lee et al., 1999).
Mitosis classification	Image processing and analysis	Pixel level likelihood (Sertel et al., 2009), Unsupervised clustering (Roullier et al., 2011), Wavelets (Weyn et al., 1998), Textural features (Irshad et al., 2013), Adaboosts (Tek, 2013).
	Machine learning	REMSS (Paul and Mukherjee, 2015), SVM (Tao et al., 2007), Genetic algorithms (Nateghi et al., 2014), AggNet (Albarqouni et al., 2016), Deep learning (Ciresan et al., 2012).

### 2.2. Challenges in Mitosis Detection and Classification

Since the development of WSI scanners, many researchers contribute with state-of-the-art methodologies to improve and integrate this technology in clinical procedures. In the academic field, a few challenges have been organized to get the attention of the scientific community and to address one of the most important issues concerning this technology: access to public datasets for validation of proposed algorithms. Although its importance, there have been only a few of these events organized in the past 6 years.

*MITOS (Mitosis Detection in Breast Cancer)* Roux et al. (2013) was the first contest organized using WSI. It was presented at the International Conference on Pattern Recognition in 2012 (ICPR 2012). The dataset released was of relatively small size (5 WSI, 10 annotated HPFs per slide) and it did not account for the inter-subject variability in tissue appearance and staining. However, the methodologies presented helped expand the literature with promising results (0.782 f-score) (Cireşan et al., 2013). To address the issues of the MITOS dataset, the *AMIDA (Assessment of Mitosis Detection Algorithms)* contest was released in 2013 and presented in the conference organized by the Medical Image Computing and Computer Assisted Intervention Society (MICCAI 2013). The dataset consisted of 23 subjects (12 for training and 11 for testing), with more than one thousand annotated mitotic figures by multiple observers. The top-performing method achieved a 0.611 f-score, and an error rate that is comparable to the inter-observer agreement among pathologists (Veta et al., 2015). At ICPR 2014, the *MITOS-ATYPIA-2014* contest was released, being organized as a follow-up and extension of the MITOS 2012 contest. The major improvements were the first nuclear atypia scoring annotations (at different nitude of magHigh Power Fields) and annotations provided by two senior pathologists (at 20x) and three junior pathologists (at 40x) (Roux et al., 2014). In 2015, the INEB (Institute of Biomedical Engineering) organized a grand challenge for its International Symposium in Applied Bioimaging (Bioimaging 2015). The objective of the competition was to classify WSI of tissue samples into four different categories: normal tissue, benign lesion, carcinoma *in situ* and invasive carcinoma. This implies that besides the analysis of nuclei, the methodologies proposed should also be able to retrieve information about the overall tissue organization. Results published in Araújo et al. (2017) showed the contribution of this challenge to the integration of deep learning algorithms into CAD systems for cancer diagnosis. Recently, in 2018, four different challenges concerning nuclei analysis in WSI were released. The *BACH (Breast Cancer Histology Image)* challenge was presented in the International Conference on Image Analysis and Recognition (ICIAR 2018) and it extended the Bioimaging 2015 challenge by incorporating a pixel-wise labeling task. Final results showed an 88% accuracy for the nuclei classification and a 66% accuracy in the pixel-wise labeling of WSI (Aresta et al., 2018). The remaining challenges were organized by MICCAI. MoNuSeg (Kumar et al., 2017), presented at MICCAI 2018, aimed at segmenting nuclei from WSI of different patients and multiple organs. A 0.6907 aggregated Jaccard index (AJI) was reported by the winning team. Meanwhile, in MICCAI CPM (2018b) the competition focuses on images extracted from a set of Glioblastoma and Lower Grade Glioma whole slide tissue images. Preliminary results in this challenge showed a 0.85 on the average of the dice coefficients. Finally, the challenge presented in MICCAI CPM (2018a) evaluates the performance of automated classification algorithms when information from two types of imaging data (i.e., radiology images and pathology images) is used. Whole slides images correspond to two subtypes of lower grade glioma tumor cases: Oligodendroglioma and Astrocytoma. No preliminary results have been reported.

## 3. Methodology

We build upon two methodologies (i.e., AlexNet and U-Net) a framework to compare both neural networks and define which is more suitable for biomedical applications, especially when dealing with Whole Slide Images and mitosis detection.

### 3.1. Dataset

Images used to evaluate the proposed methodologies were gathered from two public datasets corresponding to the ICPR-2012 contest (Roux et al., 2013), and the MITOS-ATYPIA-2014 challenge (Roux et al., 2014). Both datasets contain images corresponding to 10 High Power Fields (HPF) at 40x magnification, selected from Whole Slide Images (WSIs) by experienced pathologists. Two scanners were used to digitize the tissue samples: Aperio Scanscope XT with 1pixel = 0.2456μ*m* resolution; and Hamamatsu Nanozoomer 2.0-HT with 1pixel = 0.2273μ*m* resolution. The datasets combined added up to 2177 frames which are further processed to obtain the input patches for both neural networks. To build the masks for the U-Net, a manual segmentation of each mitosis and non-mitosis in the MITOS-ATYPIA 2014 dataset was necessary, due to the lack of ground truth for region segmentation. In addition, data augmentation was only needed for the AlexNet, as CNNs require large datasets to train. This was implemented by rotating the patches in four different angles (0°, 45°, 90°, 180°), and incorporating data from a third dataset (AMIDA13) reported in Saha et al. (2018). Patch sizes were dependent on the network configuration, and, in the case of the AlexNet, the 71 × 71-pixel size correspond to the largest mitosis found in both datasets. Table 2 summarizes the distribution of the data for both neural networks. The total number of patches differs as we used data augmentation for the AlexNet dataset.

**Table 2:** Summary of the datasets used in the AlexNet and U-Net.

**Neural network**	**Patch size**	**Mitosis**	**Non-mitosis**	**Dataset**
AlexNet	71 × 71	5850	7563	ICPR2012 & MITOS-ATYPIA2014 & AMIDA13
U-Net	128 × 128	327	0	ICPR2012
		946	3585	MITOS-ATYPIA2014

### 3.2. Color Normalization

Hematoxylin-and-eosin (H&E) staining is widely used in the histopathological analysis. However, despite more advanced and automatized staining devices (if existing) this staining still remains highly dependent on staining providers, concentration, chemical reactivity, storage conditions, and timing. These factors, combined with the fact that light transmission depends on tissue thickness as well as on mechanical and optical properties of the scanners, generate one of the most common problems when using automated image processing algorithms: the color variability (Ben Cheikh, 2017). In (Hoffman et al., 2014), Hoffman et al., reported a comparison between several color normalization methods which conceptually resembles Reinhard's statistical methodology reported in Reinhard et al. (2001). Based on the latter, the RGB channels of each frame in the dataset were transformed into the *Lab* color space, in order to modify its color characteristics following Equation (1).

(1) $$\begin{array}{l} I_{\text{n}}^{\left(i\right)}=\left(I_{\text{s}}^{\left(i\right)}-\mu_{\text{s}}^{\left(i\right)}\right)\frac{\sigma_{\text{t}}^{\left(i\right)}}{\sigma_{\text{s}}^{\left(i\right)}}+\mu_{\text{t}}^{\left(i\right)},i\in \left\{L,a,b\right\} \end{array}$$

$I_{\text{n}}^{\left(i\right)},I_{\text{s}}^{\left(i\right)}$ correspond to the normalized and the source RGB frame transform to the *Lab* color space. In addition, $\sigma_{\text{s,t}}^{\left(i\right)}$ and $\sigma_{s,t}^{\left(i\right)}$ correspond to the standard deviation and mean value computed over all the pixels of the source's and target's *Lab* channel *i* respectively. The target image corresponds to the mean image of all the dataset. After normalization, the frame is returned to the RGB color space, where each channel showed a separable color distribution when evaluating its histogram. This normalization was performed on the original frames before generating the patches.

### 3.3. Blue Ratio Computation

H&E staining presents certain characteristics which allow the automatic detection of potential mitosis inside the High Power Field (HPF). In fact, the Hematoxylin stains the cell nuclei in a blue-purple shade, while Eosin stains cytoplasm and stroma in various shades from reddish to a pinkish color, and collagen in a pale pink shade. Due to its particular blue color shade observed in the RGB image, it was reported in Irshad et al. (2013), Ben Cheikh (2017), and Saha et al. (2018) that the blue ratio image highlights nuclei as it enhances the blue color layer following the transformation in Equation (2).

(2) $$\begin{array}{l} I_{BR}=\frac{255\times B}{\left(1+R+G\right)\left(1+R+G+B\right)} \end{array}$$

*R, G*, and *B* are the red, green and blue channels respectively. After the generation of the blue ratio image, segmentation and a morphological opening operation were applied. The objective is to retain the potential mitosis present in the HPF for further analysis. The threshold and the radius of the opening operation were obtained empirically by validating them in the ICPR-2012 dataset. Testing was also performed using the MITOS-ATYPIA-2014 dataset. Using the potential candidates selected from the blue ratio image, 71 × 71-pixel patches were extracted from the original HPF. These patches were augmented afterward by rotation to increase the input data for training and testing the AlexNet. Regarding the U-Net, the blue ratio image was added as a fourth layer (i.e., R, G, B, BR) to the HPF. Then, patches of 128 × 128 pixels, centered at the centroid of a mitotic/non-mitotic cell, were extracted from each frame and its corresponding labeled mask was also generated.

### 3.4. Deep Learning Architectures

Convolutional Neural Networks (CNN) have proven to outperform classic machine learning techniques regarding image processing applications. Previously, it was reported that statistics from textural features and color features are frequently used along with different classic classifiers and CNN. In our study, the performance of the CNN proposed in Krizhevsky et al. (2012) (currently known as AlexNet) was evaluated. The input layer was modified in order to allow the CNN to work with color-normalized patches of 71 × 71 pixels. The network was also fine-tuned in order to obtain a binary classification (mitosis and non-mitosis) rather than a 1000-class classification as originally proposed for the ImageNet dataset. No handcrafted features were added to the computation and the training was performed using stochastic gradient descent, a batch size of 128 and 100 epochs. Finally, all patches generated from the BR image were divided into three datasets in the following proportions: 70% for training, 20% for validation, and 10% for testing.

A second deep learning approach (so far unreported in the literature for this application) using the U-net, was also implemented. This 23-layer network, initially reported in Ronneberger et al. (2015), was trained for 100 epochs using stochastic gradient descent, unitary batch size, Adam's optimizer, and a binary cross-entropy loss function. As it was detailed in Table 2, the patches used for the U-Net were unbalanced; therefore, we chose 898 samples (701 non-mitotic patches and 197 mitotic patches) for testing and the remaining 2884 non-mitotic patches were randomly selected to match the 1076 mitotic remaining patches. Additionally, these 2152 selected patches were again randomly divided into 90% for training and 10% for validation.

To increase the performance in terms of computational time, both neural networks were trained in a parallel platform using two Nvidia Quadro M2000 GPUs. Furthermore, the CNN was implemented using DIGITS interface from Nvidia and the Caffe framework. Meanwhile, the U-Net was implemented using Keras and Tensorflow as a backend framework for deep learning.

### 3.5. Validation Metrics

In order to measure the performance of the proposed methodologies, five metrics were selected. Table 3 relates the metrics with its corresponding dataset and neural network.

- **Accuracy**: $\frac{TP+TN}{TP+TN+FP+FN}$
- **Sensitivity**: $\frac{TP}{TP+FN}$
- **Specificity**: $\frac{TN}{TN+FP}$
- **F1-score**: $2\frac{Precision\times Recall}{Precision+Recall}$
- **Dice index**: also known as the Sorensen-Dice coefficient, is defined as $\frac{\left|Ŷ\cap Y\right|}{\left|Ŷ|+|Y\right|}$, where *Ŷ* are the predicted labeled masks, and *Y* are the ground truth masks.

**Table 3:** Metrics used to evaluate the performance of the algorithms.

**Neural network**	**Evaluation metric**	**Dataset used**
AlexNet	Accuracy	Train/test
	Sensitivity	Test
	Specificity	Test
	F1-score	Test
U-Net	Accuracy	Train/test
	Dice index	Train/test

## 4. Results and Discussion

### 4.1. Detection of Potential Mitosis

**Figure 1:**
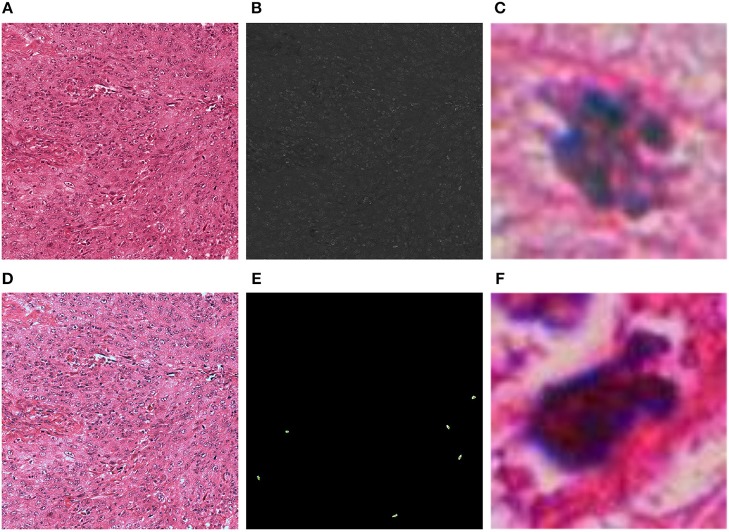
Results of the color normalization algorithm and blue ratio image generation for one frame: **(A)** Original HPF; **(B)** Blue ratio (BR) image; **(C)** Mitotic 71 × 71 patch generated with the thresholded version of the BR image; **(D)** Color normalized HPF; **(E)** Detection of mitosis centroids using the BR image to validate true positive rate; **(F)** Non-mitotic 71 × 71 patch generated with the thresholded version of the BR image.

A critical step in the CNN approach using the AlexNet is the detection of potential mitosis in the HPF frames. Figure 1 resumes the results for the color normalization algorithm and blue ratio image computation. The threshold to generate the binary image from the BR image and the radius of the structure used for the opening operation were found to be *T*_*h*_ = 30, and *R*_*d*_ = 1, respectively. These parameters allow achieving a 100% detection of true positive mitosis in the validation dataset (ICPR2012). Additionally, when tested in the MITOS-ATYPIA2014 dataset, the true positive rate was approximately 0.99, meaning only 1 mitosis was not detected from the whole dataset. Regarding the semantic segmentation approach, the RBG input was normalized without any further preprocessing steps.

### 4.2. Convolutional Neural Network (AlexNet)

**Figure 2:**
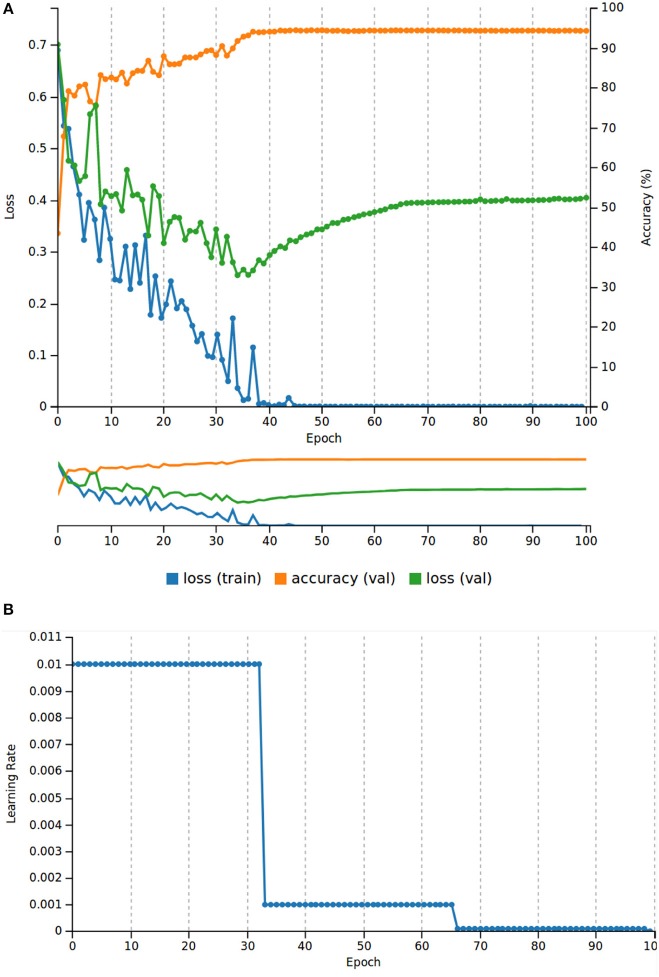
Results of the AlexNet training. **(A)** Training and validation loss values, and accuracy for the validation dataset (20% of the total patch dataset). **(B)** Learning rate behavior for 100 epochs.

Figure 2 resumes the results throughout the entire training and validation as well as the learning rate behavior. After 100 epochs of training, we observed a descending behavior of the loss value until epoch 40. At epoch 100, the loss value for the validation was only 0.1 higher than the one at epoch 40; however, this steady increase of the loss function indicated that the AlexNet was starting to overfit at epoch 40. To overcome this issue, we used the weights saved in epoch 40 to test the performance of the network in the test dataset. Regarding, the validation dataset and before the CNN started to overfit, the network showed a performance of 94.35% of accuracy.

The CNN was also evaluated using a testing-patch dataset which was never considered in training, nor in the validation process. Table 4 shows the confusion matrix computed with the testing patch dataset. Additionally, Table 5 shows a resume of the metrics defined to evaluate the performance of this CNN.

**Table 4:** AlexNet: Confusion matrix for the testing patch dataset.

	**Mitosis**	**Non-mitosis**	**Accuracy/class**
Mitosis	551	34	94.19%
Non-mitosis	32	724	95.77%

**Table 5:** AlexNet: Evaluation metrics in the testing patch dataset.

**Accuracy**	**Sensitivity**	**Specificity**	**F1-score**
95.08%	94.19%	95.77%	94.35%

### 4.3. Semantic Segmentation (U-Net)

**Figure 3:**
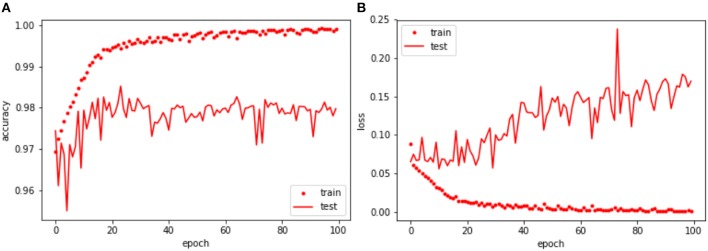
U-Net: Training and validation throughout 100 epochs. **(A)** Training and validation accuracy of each epoch. **(B)** Training and validation loss value of each epoch.

**Figure 4:**
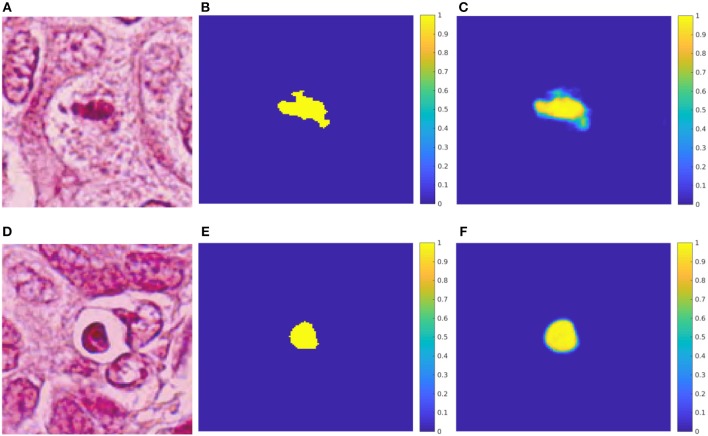
Test results for the U-net. **(A)** HPF patch #37 with a mitosis. **(B)** GT mask of the mitosis. **(C)** Predicted mask for the mitosis class. **(D)** HPF patch #58 with a non-mitotic cell. **(E)** GT mask of the non-mitosis. **(F)** Predicted mask for the non-mitosis class.

**Figure 5:**
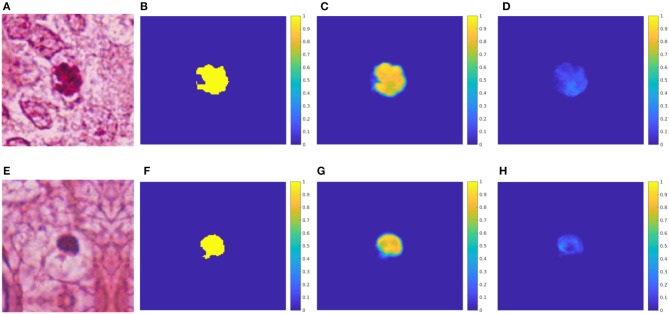
U-net: Probability maps for both predicted classes in the testing-patch dataset. **(A)** HPF patch #35 with a mitosis. **(B)** GT mask of the mitosis. **(C)** Predicted mask for the mitosis class. **(D)** Predicted mask for the non-mitosis class. **(E)** HPF patch #128 with a non-mitotic cell. **(F)** GT mask of the non-mitosis. **(G)** Predicted mask for the non-mitosis class. **(H)** Predicted mask for the mitosis class.

Table 6 and Figure 3 resume the performance of the network after 100 epochs of training. The overall accuracy was 97.98% in the validation patch dataset, and 97.73% in the testing patch dataset. Regarding the convergence using Adam optimizer, the loss value for both training and validation dataset showed a decreasing behavior. Similarly to the AlexNet, early stopping of the network was applied around epoch #24 due to the constant average loss value in the validation dataset and the increasing behavior afterward. In addition, the Dice coefficient, obtained in both validation and testing datasets, indicates that nearly 60% of the predicted mask overlaps with the ground truth which translates into good network performance. In Figure 4, two RGB patches, ground truth masks, and predicted masks corresponding to mitosis and non-mitosis are shown. We found that almost every pixel corresponded to the ground truth classification, with the exception of the borders which were not well defined in the predicted masks. Additionally, in certain cases, the classification is not 100% accurate and a low probability map (i.e., predicted mask) is produced by the neural network as seen in Figure 5.

**Table 6:** U-Net: Evaluation metrics in training and testing.

**Dataset**	**Train**	**Validation**	**Test**
Dice index	0.9747	0.6117	0.5842
Accuracy	99.90%	97.98%	97.73%

### 4.4. Discussion

The blue ratio has proven to be an efficient algorithm to detect potential mitosis with excellent accuracy (almost 0% false negative error). The analysis of the HPF containing the missed mitosis showed that the error presented when the WSI was scanned with the Hamamatsu scanner. This may suggest that the difference in resolution among scanners is likely to impact the quality of the algorithms used to preprocess the data. Additionally, commercial scanners perform a series of focusing actions during the digitalization of a tissue sample. These changes in the focus—due to fine mechanical slide scanners limitations—seem also likely to impact the quality of the whole slide image generated and, implicitly, of the analysis algorithms used for computation.

**Figure 6:**
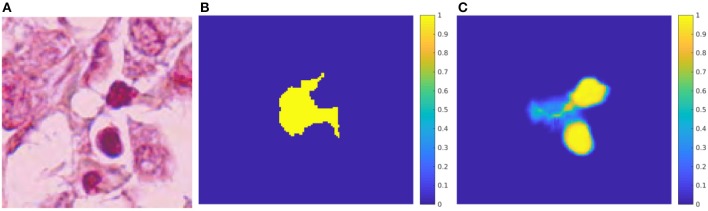
Evaluation of SLIC for mitosis selection. **(A)** HPF patch #75 with a non-mitosis. **(B)** Mask of the mitosis generated using SLIC. **(C)** Predicted mask for the mitosis class using U-Net.

Although BR obtained high accuracy, it was computationally time-consuming due to the generation of false positive cases. To generate the nearly 13000 patches used in this work, approximately 2.5 h were needed using a Core i7 processor computer with 24GB of RAM. To increase performance, both deep learning approaches were optimized to run in NVIDIA GPUs. The AlexNet, on a GeForce GTX980M, only took 20 min and 50 s to train, and 5 s to test nearly 1200 images. Meanwhile, the U-net, on two Quadro M2000, was trained in approximately 5 h, and testing took 46 s per 128 × 128 pixels patch. The second deep learning algorithm presented in this article, based on the U-net, represents an approach closer to an end-to-end deep learning architecture. The complete elimination of handcrafted features (e.g., BR images) made the solution more robust to color and textural changes, as observed in the slight increase of accuracy, by opposition to AlexNet. Compared to the results in the literature, metrics obtained from both neural networks validate the fact that handcrafted features might introduce errors and subjectivity into the classification. To address this issue, we generated ground truth masks for the testing dataset using a Simple Linear Iterative Clustering (SLIC) algorithm to create super-pixels in a HPF frame which correspond to mitotic or non-mitotic clusters of pixels. Figure 6 shows an example of a non-mitotic cell. The mask predicted using the SLIC algorithm involves several pink pixels from the patch which does not correspond to the cell; meanwhile, the mask generated by the U-Net clearly identifies the region with predominant blue coloration representing the presence of a cell, in this case, non-mitosis. Throughout the entire testing dataset, we found similar cases which strengthen the hypothesis that hand-crafted features introduce errors in the detection and classification of the behavior of cellular structures.

The results in the present work outperform all classical machine learning approaches existing at the moment and reported in the literature review section. However, for the U-net, further analysis is needed in order to improve border detection (increasing of Dice index) and increase the sensitivity close to 100%, essential in biomedical applications. This study presented two methods to analyze and classify a frame containing 10 HPF, usually observed by pathologists. Due to the structure of the networks and its performance, these algorithms can also be applied directly to the WSI. The direct application to WSI—which we will study in our future research—will improve the diagnosis of breast cancer as pathologists only evaluate small regions of the sample tissues.

## 5. Conclusions and Perspectives on Computational Pathology

In this article, we have shown how two different deep learning approaches (i.e., AlexNet and U-Net) performs when dealing with the classification and detection of mitosis. The results suggest that the performance of classical image-processing methodologies and deep learning approaches, combined with hand-crafted features, can be noticeably improved (nearly 7% improvement in accuracy respect to the last work in Saha et al., 2018). Regarding the AlexNet, we found that it is possible to adapt the neural network to work with small patches corresponding to single mitosis. Although this method has the disadvantage of using a pre-processing step (i.e., BR computation to detect potential mitosis), it outperforms the proposed algorithms in the literature.

On the other hand, due to the semantic advantage of the U-Net, we can directly detect (finding the *where*) and classify (finding the *what*) cellular structures in an end-to-end framework without the need of pre/post-processing steps (e.g., the AlexNet needs the BR to find potential mitosis and to obtain context information we might need to follow the sliding window approach which is not computational-friendly). This suggests that the U-Net is more suitable and robust to process WSI allowing a stable and effective transition toward the full WSI analysis. Although the U-Net does not depend on the size of the input image, WSI analysis may turn out a greater challenge due to its size, different cellular patterns and structures, and artifacts introduce by scanners, staining process or other human factors. Therefore, future research to validate the use of deep learning architectures (such as the U-Net in WSI) is needed.

We have seen that the U-Net is an excellent tool to analyze histopathological images (validated in HPF so far). Therefore, we can extend the solution and apply it to different nuclei detection such as neoplastic nuclei, fibroblast, lymphocyte, adipose tissue (adipocytes), stroma, or blood vessels. These nuclei allow pathologists to have a better understanding of the tumor microenvironment which benefits the patient in terms of targeted treatment to specific characteristics observed in the tumor. Additionally, we can also use the U-Net to analyze the spatial distribution of the tumor microenvironment, in order to further understand tumor heterogeneity which might, in turn, provide some insight on why certain types of cancers are more resistant than others. Tumor heterogeneity can manifest as intra-tumor (meaning clones of cells responding differently to the same treatment), or inter-tumor (meaning, same kind of tumor behaving differently in different patients). In both cases, the semantic property of the U-Net allows to have a pixel-wise understanding of the tissue and therefore give the pathologists and oncologists useful information to diagnose and treat their patients.

Finally, in this work, we used images of breast cancer tissues. However, this can be applied to all types of tissue as the deep learning architectures can be easily adapted to other inputs.

## Author Contributions

All authors contributed to the methodology proposal, analysis of results, writing, and review of the manuscript.

### Conflict of Interest Statement

The authors declare that the research was conducted in the absence of any commercial or financial relationships that could be construed as a potential conflict of interest.
